# Central Dental Association of Northern New Jersey

**Published:** 1892-02

**Authors:** 


Adjourned.

---

### CENTRAL DENTAL ASSOCIATION OF NORTHERN NEW JERSEY.

THE regular meeting was held in Newark, N. J., December 15, 1890.

*The President.*—Gentlemen, we will now listen to Dr. Westlake, who will read the paper of the evening, entitled "Ethical Environments of the Dental Surgeon."

(For Dr. Westlake's paper, see page 36.)

### DISCUSSION.

*Dr. Stockton.*—The environments of the "dental surgeon" are many. The incidents mentioned by the essayist, perhaps, are not the least. As a rule, I have ever found the physician quite willing to defer to that which clearly and properly belongs to the dentist. I have known of some few instances when the errors of the physician have been quite disastrous; but we all know little enough, and I think the practice of the day among all intelligent physicians is that they shall do what they are educated and trained to do, and that the dentist shall likewise do what he is educated to do.

The need of an education is one of the most prominent factors in the problem. Men complain of the want of appreciation, and blame the world for not giving them recognition and success, but the fault is at their own door, and they have entered upon the duties of what they are pleased to call a profession, while it may be they are only prepared for the wants of a trade.

The young man entering the profession through our State laws, may think it somewhat of a hardship, and a work of supererogation, to be compelled to pass another examination, fresh from college. Yet, in after years, he will look back and date the beginning of his manhood from this event in his existence, provided always the Board of Examiners do him full justice.

There is no hardship in being compelled to know all that is available in the beginning of our professional or business career. The wheel of life whirls round, and we whirl with it, expecting that the motion will some day slacken, and that then omissions may be made good. But real wisdom consists in packing your storehouse before the winter of discontent sets in.

We have a code of ethics governing us in our relations to each other; rules for our conduct towards each other; and if we transgress them, we are held responsible.

But other duties come in, and they are not all on one side. The ethical relation extends far beyond this, and has its obligations to those and from those for whom we perform our daily labors. The ethical is something that can be appealed to in order to keep peace and harmony amid the conflicting interests of men, to help forward social welfare, and in this way reacting to the benefit of the individual. It is then a sort of police ordinance, written originally in the soul or impressed thereon by society itself, because experience has shown the necessity of it. Ethics may be a science, since it aims to exhibit systematically the facts of moral nature and the principles of conduct. It may be a philosophy, since it aims to set forth the ultimate assumptions in which all these facts and principles have their origin.

Then the end to be sought in life may be learned in general by the study of the nature of man as related to the sphere in which he lives, or is to live, just as we learn the ends of other things by studying the structure as related to their sphere.

But we have not yet a complete science of human nature and relations. Rules of life are thus far subjects for the exercise of good judgment and common sense rather than strict scientific definition.

Resentment against wrong and injury is a sort of natural or implanted moral tone on the side of justice, and when resentment is moralized into justice and the kindly affections into benevolence, it is often thought that the whole field of ethics is covered.

Let us see, to-night, how this ethical relation acts in our every-day practice.

For instance, the other day a lady came to the office, and her salutation was, "Doctor, the tooth you put in for me a short time since has broken in pieces; it broke on my way to market this morning."

I said, "Madame, your tooth did not break itself."

Her reply was somewhat startling,—"Do you think I tell a lie?"

Another tooth was prepared, and as I was about to insert it I handed her a glass, and asked her to see that it was perfect in all respects. She declined the examination, saying it might have a crack *inside*, which she could not see.

Again, you are engaged operating for, it may be, some very particular or distinguished patient, who may have come to you because she has heard your fillings never come out, and in walks a patient, whose first exclamation may be, "Doctor, the filling you put in my tooth has come out." You kindly ask her to take a seat and that you will see her soon. You finish the operation for your distinguished patient, and maybe suggest another appointment, as other work is needed, but she has heard the remarks about the filling, and will not make it now. The patient with the filling out takes the chair, and, after a careful examination and reference to your record-book, you find everything intact, but that a new cavity, in an adjoining tooth perhaps, has just made itself manifest, and you received the blame.

So the subject of the paper, the ethical environments of the dental surgeon, comes home to us in one way or another, and is one that we may and should carry with us, and the thought may arise during its contemplation that we should do unto others as we would they should do to us under the same circumstances.

*Dr. Osmun.*—One of the points in the paper I have spoken of before,—that is, the education of the public. We hear a great deal about the education of dentists and the elevation of the dental profession, and that is all right and proper; but there is another thing for the dental profession to do, and that is to take steps to educate the people, so that they can understand what the dental profession is doing. They live in almost utter ignorance of what is being done in the dental ranks. They take the standard of their forefathers and apply it to the advanced dental science of to-day. It is the duty of the dental profession, through its societies, the papers, and the magazines, to spread before the public what is being done in dentistry; and if that were done systematically and thoroughly we would not hear so many remarks and criticisms of the kind that were detailed by the essayist and the preceding speaker.

In reference to the relation of physicians to dentistry, I must say I have never had any of the experience spoken of. The physicians of Newark have treated me, and I think all the dentists of standing, with the greatest consideration, regard, and respect. I have never had any occasion to complain of these things that we hear so much about. I think they are learning a lesson that dentistry is not a trade.

*Dr. Sanger.*—To one who listens only to the reading of a paper its full value is lost, because the argument, however plain the writer tries to make it, cannot but be somewhat subtile. He has a thought in his mind which he is trying to convey to us, and his words and his thoughts are not our thoughts and words; our ideas do not run parallel with his, and we do not fully catch his meaning. After a paper is printed, and we read it, we are surprised to find a language and force of argument that we did not notice when the paper was read. Dr. Stockton had that advantage, so he could discuss this paper intelligently. The idea suggested by Father Atkinson, that copies of papers to be read before societies should be sent to different members, in order that they may prepare themselves for the discussion of them, is to my mind a good idea, and I think the time will soon come when we shall have such copies printed and placed in the hands of those whom the essayist may designate.

The older school of physicians especially need a little lifting up on the line of dental surgery. We do meet constantly cases where good dental surgery is needed and very bad surgery is practised, and teeth are lost because they fell first into the hands of medical men. It remains for us to educate these men. They are not going back to college and begin over again, and read text-books on the science of dental surgery. It is our duty and privilege to lift up our profession in every way, by practising dental ethics towards each other, the medical profession, and our patients, so that when they come into our hands we shall not take advantage of the fact that some other practitioner has perhaps made a mistake and has been unfortunate enough to make a bad operation, but we should excuse the fault, remembering that we may make the same mistake, and repair the damage, if there be any, and send the patient out of our office with a kindly word for the man who did the work before us.

*Dr. G. Carleton Brown.*—I have to disagree a little with the essayist in one or two points. One is the idea that we are a chosen people. I do not believe it. We are what we make ourselves, and not a chosen people. Another point is that the medical profession is all wrong and the dental profession all right. I do not believe it. I think we can make mistakes. They have things to learn from us, perhaps, and we have things to learn from them; and I am of the opinion that when we come to examine the ethical environment of the dental surgeon we will find just as much on the other side of the question as on the side that has been presented to-night.

*Dr. Ottolengui.*—Mr. President, the point is this: we have been recognized. We are now a branch of medicine; dentistry is a specialty of medicine, and we are considered by medical men as specialists. The man who is a regular physician knows all about obstetrics, but the gynæcologist knows a little more than he does, because he has more practice in that special field; but dental literature, which is so nearly analogous to medical literature, medical men do not know anything about. I will relate a little incident that occurred recently to illustrate what I mean. When I first became associated with Dr. Kingsley, a lady was passed over to me who was wearing an obturator. This patient had a fissure so large that you could put the fist of a baby in the hole, the turbinated bones were very much atrophied, and but little of the tissue of the soft palate was left. That patient when she first came into Dr. Kingsley's hands was only fourteen years old. She wore a velum for seven or eight years, and was most carefully instructed in articulation and speech, until she could do without the velum, then was given an obturator. Ten years after that she was given a new obturator; the case covering about twenty years since she first came for the help of an appliance. There is now nothing the matter with her speech. Ever since she was first placed in my hands she has been practically my patient. Recently the obturator was broken, and so I suggested that it be repaired. She said she had been considering the matter, and had been advised, and would not do anything without Dr. Kingsley's permission. I said, "What have you been advised to do?" She said, "I have been advised by a great specialist of New York to have this fissure sewn up; he says this thing is well enough in its way, but it is one of those things that a mechanic makes to tide over a case he does not know how to control." That gentleman you would all know, if I should mention his name; he is one of the celebrities in New York. This is one case. There is a child living here in Newark whose parents were so anxious to have a cleft palate remedied that the child was brought to Dr. Kingsley when it was two years old, to know whether the fissure could be sewn up. He said, Yes it could, but his experience was that it never did any good, except perhaps in some cases where there was a great amount of tissue. But they thought they would try it, and they went to a great specialist in New York and had the operation performed. If you know what that means, you know it means a long time of trial and tribulation for the little baby, from three to five weeks of misery. In this case it was longer; and after it had gone on for four or five weeks the fissure reopened, and it was sewn up again. That time the stitches held and the child was pronounced cured. Three months afterwards the cleft opened all along the line of the hard palate, and it was left one of the worst kind of cases to treat. It is easier to fit an obturator for any other fissure than one that has been sewn up. That child shows the failure of staphylorrhaphy most signally. No man here could understand a word that that baby said last summer. An instrument was made for her, and she has been trained carefully in articulation. She is brought over every three months, and every time she comes she speaks fifty or sixty per cent. better than before. These are successes that are being made by dentists in remedying these things; and yet I can show you clippings from the medical press, from one of the first scientific journals of Europe, condemning that very class of treatment, and saying that dentists tell of the wonderful improvements they have made, when they do not know what they are about. I do not believe the person who wrote that ever saw such an operation or knows anything in regard to it.

A gentleman came in the other day and said he had heard that Dr. Kingsley had some experience in treating cleft palates, and he would like to know what to do; that he had a baby born to one of his patients with a cleft palate and he felt concerned about it. I said, "Is there a cleft in the lip?" He said there was. I said, "You had better take care of that; give the child as much lip as you can, but do not crush the maxillary bones." In one case where that was done the result was that the centrals and laterals were removed and the two canine teeth are in the centre of the mouth, and the jaw is so collapsed that it looks as though the patient had the highest kind of a roof, and the lower lip sticks out half an inch beyond the upper lip. There is no possibility of making a man articulate with that kind of lip. That is what the surgeons are doing right along in this class of cases, and they think they have fixed it all right.

*Dr. Westlake.*—I have among my patients four physicians, and they are intelligent men, and I know a great many physicians who are intelligent men and who believe a great deal in dental surgery, but they are exceptions. A physician, well-known in Elizabeth, and connected with the hospital, was in the same car with me recently. I said, "Do you have many oral surgical cases?" "Oh, yes; quite a number." "Do you have your dental surgeon perform these operations, as they do in some hospitals?" "No; what good is a dental surgeon except for pulling out teeth? If I had an exsection or a resection of the jaw I would not have a dental surgeon anywhere near me. I would not allow a dental surgeon to operate in the hospital." Now, I think, the public should be educated in this matter.

*Dr. Watkins.*—I have enjoyed these papers and the discussion very much. The statement in regard to hospital physicians reminds me of a case which fell into my hands some years since. The wife of a homœopathic physician came to my office, and her husband with her, to consult me about her teeth. I looked through her mouth and began to give advice, recommending amalgam fillings. The husband said, "Stop right there. We will have no amalgam." But I insisted on amalgam, and I carried my point. I succeeded in putting in fifteen large amalgam fillings, and she has never been salivated yet.

Articles are appearing from Sunday to Sunday on different subjects pertaining to dentistry in the *New York Herald*, generally a column, and sometimes three or four columns, each time taking up a different subject, and treating it so thoroughly as to give the public a very fair idea of the matter. Since these articles have been appearing in the *New York Herald* patients have come into my office speaking intelligently in regard to devitalizing pulps, removing pulps and putting on crowns, bridge-work, and implanting teeth; and when I would make a proposition to perform a certain operation they would fall right into line with me, and I would have very little difficulty in convincing them that what I proposed was the right thing to do.

*Dr. G. Carleton Brown.*—Dr. Westlake made some remarks about a physician of Elizabeth, or, in other words, a most eminent physician, of foreign extraction, connected with one of our hospitals. Now, it happens that I am a dental surgeon of the Elizabeth Hospital, and I will say that there was a case of fracture of the jaw for which I made a splint some time ago, or, at least, Dr. Pinney made the splint, as I wanted to see his method that he used in the Newark Hospital. He made it, and I put it on later, and it worked nicely, but one of the physicians insisted on tearing the bandages off and taking the splint out. A surgeon has charge of a case during his term of office only, and this passed from one to another, and happened to get into the hands of this gentleman of foreign extraction, and he decided that the best thing to do with that fractured jaw was to cut it out. He cut it out. There was some slight necrosis about the fracture.

I do not say that physicians do not make mistakes, but I do not think there is a living dentist who would approve of the excision of the jaw under those circumstances. There was another case where a man was struck in a boiler explosion and sustained a bad fracture of the jaw. I made a splint for him, and the splint was working beautifully, but he was taken to the hospital, and there this same surgeon saw him, and he immediately proceeded to take the splint out, asserting that the jaw would take care of itself. But he is an exception. I do not feel he should be held up as a fair representative of the medical profession of Elizabeth. I think all the dentists there will agree that, with one or two exceptions, we are consulted by the medical practitioners in all cases where we should be consulted.

*Dr. Atkinson.*—I have too much to say to even begin to say what I would like. It is a question of such deep interest to the human race, and especially to healers of all grades, that we cannot but feel interested whenever the subject is broached, but it is our false morality that has held the ethical field against mankind generally, and it is next to impossible to tell the truth without offending everybody you speak to. It is very easy for dentists to find fault with the men who have assumed a knowledge they did not have, and did not even have sufficient knowledge to entitle them to opinions about the cases they presume to manage. I have been an advocate of dentists having charge of all conditions of the mouth. My complaint is this: that men are not broad enough nor truly in love with the truth themselves to pity the men who assume a knowledge they haven't got, and forget that they themselves have done the very same things they condemn, when they had but little ground to claim that they were masters of the situation. If I were forced into the position of saying whether I would select a physician or surgeon from the medical men at large or take a dentist, as they run, I would go for the dentist. They are dealing with demonstrative things, and if they begin at a low standard they are bound to rise to a higher and a better appreciation than the men who are everlastingly dealing with figments of fancy, and following the dictum of men who have charge of the poor wretches who go into the hospitals. If we had sufficient juridical balance and mental affection to consider the subject before us and hear whatever testimony there is, and to distinguish between what is really a performance and that which is professed, we would make progress.

You have often thought I was severe upon those who confined themselves to the text-books and hospital practice. If what I said was true, then it was severe; but it was not my affection that was severe, it was my intellection that was severe. But I have been very lenient in telling the truth against the men who claim to be masters of the situation. Who has advocated dental hospitals? Who has opened his office to everybody, the ignorant and the intelligent, and asked them to come and see and criticise? The angels did. They lead every soul that loves the truth to do the things that are requisite for the redemption of the individuals who have been brought into their uncomfortable condition by reason of following a false faith.

*The President.*—Now, if Dr. Westlake has anything to say, in closing, we will listen to him.

*Dr. Westlake.*—I have nothing to say, except to thank the society for their attention.

*Dr. Atkinson.*—If you wish to get the latest investigations on that subject, you will consult the writings of a German, Dr. Zeus, who has made a series of observations in this line. You can learn how to get the book by calling upon Dr. Bödecker.

